# Neuroimmunomodulation in Major Depressive Disorder: Focus on Caspase 1, Inducible Nitric Oxide Synthase*,* and Interferon-Gamma

**DOI:** 10.1007/s12035-018-1359-3

**Published:** 2018-10-10

**Authors:** Antonio Inserra, Claudio Alberto Mastronardi, Geraint Rogers, Julio Licinio, Ma-Li Wong

**Affiliations:** 1 Mind and Brain Theme, South Australian Health and Medical Research Institute, Adelaide, Australia; 2 Department of Psychiatry, College of Medicine and Public Health, Flinders University, Bedford Park, Australia; 3 Centre for Neuroscience, Flinders University, Bedford Park, Australia; 4 School of Medicine and Health Sciences, Universidad Del Rosario, Bogota, Colombia; 5 Neuroscience (NEUROS) Research Group, Universidad del Rosario, Bogota, Colombia; 6 Infection and Immunity Theme, South Australia Health and Medical Research Institute, North Terrace, Adelaide, SA Australia; 7 SAHMRI Microbiome Research Laboratory, Flinders University College of Medicine and Public Health, Bedford Park, SA Australia; 8 College of Medicine, State University of New York Upstate Medical University, Syracuse, NY USA; 9 Department of Psychiatry, State University of New York Upstate Medical University, Syracuse, NY USA

**Keywords:** Major depressive disorder, MDD, Inflammation, Neuroinflammation, Caspase 1, Inflammasome, T-helper 1 (Th1), Interleukin 1, Inducible nitric oxide synthase, Interferon gamma, Gut microbiome

## Abstract

Major depressive disorder (MDD) is one of the leading causes of disability worldwide, and its incidence is expected to increase. Despite tremendous efforts to understand its underlying biological mechanisms, MDD pathophysiology remains elusive and pharmacotherapy outcomes are still far from ideal. Low-grade chronic inflammation seems to play a key role in mediating the interface between psychological stress, depressive symptomatology, altered intestinal microbiology, and MDD onset. We review the available pre-clinical and clinical evidence of an involvement of pro-inflammatory pathways in the pathogenesis, treatment, and remission of MDD. We focus on caspase 1, inducible nitric oxide synthase, and interferon gamma, three inflammatory systems dysregulated in MDD. Treatment strategies aiming at targeting such pathways alone or in combination with classical therapies could prove valuable in MDD. Further studies are needed to assess the safety and efficacy of immune modulation in MDD and other psychiatric disorders with neuroinflammatory components.

## Introduction

Major depressive disorder (MDD) is a psychiatric disorder with significant morbidity, mortality, disability, and economic burden worldwide [1, 2]. In addition to the psychosocial and psychophysical dysfunctions associated with MDD, several conditions are often comorbid, including but not limited to obesity, type-2 diabetes, heart conditions, autoimmune diseases, neurodegenerative disorders, cancer, and intestinal conditions [3–7]. Multiple hypotheses have been formulated attempting to describe the elusive pathophysiology of MDD, including the monoamine hypothesis, the neurotrophic hypothesis, the glutamate hypothesis, the cytokine (or macrophage) hypothesis, and the microbiota-inflammasome hypothesis [8–13]. However, no single hypothesis seems to fully explain the onset, course, and remission of the disease. To complicate matters further, antidepressant drugs present numerous side effects and are effective only in a subset of patients [14–16]. Newer therapeutic strategies involve drugs acting on neuroplasticity-related pathways, gut microbiome modulation, and deep brain stimulation surgery [17–19]. Nevertheless, the quest for a better understanding of the molecular underpinnings of this disease represents an essential step in the identification of efficacious therapeutic strategies that could target the causal biological mechanisms of MDD.

Emerging evidence suggests that dysregulated neuro-immune pathways underlie depressive symptomatology in at least a subset of MDD patients [2, 20–25]. Three crucial inter-linked networks seem to influence the bidirectional communication between the brain, the immune system, and the intestinal microbiome, namely (a) increased oxidative stress, driven by nitric oxide (NO) overproduction, (b) chronic inflammation, driven by caspase 1 (CASP1), and Nod-like receptors family pyrin domain containing 3 (NLRP3) inflammasome over activation, and (c) central nervous system (CNS) T cell-helper 1 (Th1) lymphocyte infiltration, driven by interferon-gamma (IFNG). These three networks are strictly interlinked and present several levels of reciprocal regulation. For example, NO is a critical negative modulator of the NLRP3 inflammasome, while being necessary for IFNG-mediated suppression of interleukin-1 beta (IL1B) processing [26, 27]. Moreover, CASP1 regulates IFNG production via producing IL18, while IFNG modulates the CASP1 system [28]. Similarly, transcription of inducible nitric oxide synthase (NOS2) can be activated by IFNG [29]. Lastly, CASP1 is involved in the epigenetic regulation of NOS2 [30]. These multidirectional interactions suggest the importance of observing and therapeutically approaching these pathways as a whole rather than as insular entities. The possible involvement of these three systems in MDD is briefly summarized here and will be described in detail throughout this review.

Reactive oxygen species (ROS) are produced during cell metabolism, and are largely quenched by the endogenous antioxidant machinery [31]. However, excess of oxidative products can elicit oxidative stress and cause protein, lipid, and/or DNA damage [32]. Preclinical and clinical studies suggest that chronic stress exposure is associated with increased ROS production [33–40]. One of the free radicals produced during psychological stress is NO, mainly by NOS2 [41]. Inflammatory factors play key roles in tissue repair and in defense against pathogens [42, 43]. However, pathological activation of inflammatory cascades caused by stress and other insults can alter brain function and increase the likelihood of developing MDD and comorbid conditions [44–46]. CASP1, a protease that in the NLRP3 inflammasome renders the mature forms of IL1B and IL18, is also activated by stress [47, 48]. It has been shown that reactive T cells infiltrate the brain where they produce pro-inflammatory cytokines in response to CNS antigens [49]. Lastly, IFNG is a powerful inducer of indoleamine 2,3-dioxygenase 1 (IDO1), which degrades tryptophan increasing kyneurine and quinolinic acid, leading to hyposerotonergia and hyperglutamatergia, involved in MDD [9, 50, 51].

Recently, the role of the gut microbiome in mental health and illness has come to the forefront in psychiatry [52, 53]. Increasing evidence suggests the existence of a gut-brain-axis, a communication network that integrates brain and gut function, which plays a fundamental role in health and disease [54]. Such communication occurs via the endocrine and immune systems, the vagus nerve, and the bacterial metabolome [55–57]. It is becoming clear that the gut-brain-axis is an entity directly involved in modulating stress systems like the hypothalamic-pituitary-adrenal (HPA) axis, via its effects on the immune and endocrine systems, which affect behavior and mood and that can lead to MDD [53, 58, 59]. Given its central role in modulating immune processes and brain function, and given that MDD is characterized by altered gut microbiome composition, consensus is growing that manipulating the gut microbiota could represent a therapeutic tool in the treatment of MDD [19, 60]. In this review, we will summarize the pre-clinical and clinical evidence supporting the involvement of CASP1, NOS2, and IFNG in the pathophysiological processes underlying depressive symptomatology.

## Communication Between the Brain, the Immune System, and the Gut Microbiome

Although the CNS is considered to have its “own” immune system, independent from the peripheral immune system, it is accepted that the two constantly communicate and cooperate, that the CNS is involved in regulating immunity, and that immune responses in the periphery lead to behavioral changes [66, 67].

Stress-mediated upregulation of pro-inflammatory cytokines [such as IL1, IL6, tumor necrosis factor (TNF), and IFNG] leads to endocrine and neurochemical responses, such as sympathetic nervous system (SNS), hypothalamic-pituitary-adrenal (HPA) axis, and microglial activation. SNS stimulation triggers epinephrine and norepinephrine release in the locus coeruleus and adrenal medulla, which result in an upregulation of pro-inflammatory signaling. SNS activation in response to stress pushes the CNS to “steer” immunity towards pro-inflammatory and antiviral responses [23]. At the same time, norepinephrine modulates pro-inflammatory cytokines transcription via beta-adrenergic receptor stimulation [68].

This leads to HPA axis activation by hypothalamus-secreted corticotropin releasing hormone (CRH) and arginine vasopressin (AVP). CRH stimulates adrenocorticotropic hormone (ACTH) release from the pituitary gland, which stimulates glucocorticoids release by the adrenal gland. Glucocorticoids interact with the glucocorticoid receptor (NR3C1) and the mineralocorticoid receptors (NR3C2), activating anti-inflammatory cascades and inhibiting Th1-driven pathways. This upregulates anti-inflammatory gene expression to avoid side effects [69–73]. The gut microbiome modulates HPA axis processes. In fact, germ-free rodents have greater plasma ACTH and corticosterone spikes compared to wild-type in response to stressors, while displaying altered anxiety-like behavior [74]. This exaggerated response can be reversed by early stage (but not later stage) recolonization with *Bifidobacterium infantis* [74]. Interestingly, the brain regions presenting the highest concentrations of pro-inflammatory cytokines are the prefrontal cortex, the hypothalamus, and the hippocampus, areas involved in cognition, mood, and antidepressant response [75, 76].

Increased concentrations of brain cytokines trigger the activation of microglia, immune cells inhabiting the brain parenchyma, representing chief innate immune cells in the brain [67, 77]. Depending on the temporal and qualitative cytokine profile, stress-induced microglial activation can either stimulate neuroprotection or neurodegeneration [78]. Not surprisingly, the gut microbiome modulates microglia homeostasis and maturation, while reduced gut microbiome complexity impairs microglia function [79]. Altogether, these stress-induced inflammatory events alter neurotransmitter systems, such as serotonin (5HT) and dopamine (DA), exacerbating depressive symptoms [80, 81]. Interestingly, the gut microbiome is also involved in neurotransmitter modulation, either via producing neurotransmitters, consuming them, or responding to them [82]. This raises the intriguing possibility that by altering gut microbiota composition, it might become possible to modulate neurotransmitter systems in pathological states, including MDD (Reviewed by [82]).

Glucocorticoids have the effect of restoring homeostasis [83]. However, in MDD, the HPA axis can become hyperactive. This phenomenon is underlined by increased cortisol, blunted ACTH response to CRH, glucocorticoid resistance, impairment in gluco- and mineral-corticoid signaling, and enlargement of the pituitary and adrenal glands [84–88]. Antidepressant drugs normalize the HPA axis and enhance the expression and function of corticosteroids [89, 90]. Peripheral cytokines can cross the blood-brain barrier (BBB) via (a) CNS lymphatic vessels, (b) active transport and a leaky or compromised BBB, (c) crossing at circumventricular organs, and (d) binding to receptors in the blood vessels that course through the brain [91–94]. Moreover, cytokines can affect brain function indirectly, through vagal nerve activation or by binding to cell-surface proteins found in brain endothelial cells [91, 93, 95, 96].

Cytokines can be produced in the gut in response to bacterial virulence factors (such as LPS), and in response to bacterial translocation to physiologically sterile enteric compartments (“leaky gut”) [97]. It was proposed that the leaky gut phenomenon contributes to MDD [98]. In fact, stress is known to compromise gut epithelial barrier integrity, allowing gut bacteria to access the enteric nervous system and immune cells [99]. Intestinal inflammation is a major contributor to changes in gut microbiome composition and function that are associated with disease (Reviewed in [100]). IFNG triggers the production of hydrogen peroxide and the epithelial expression of NOS2, which elevates the concentration of NO, in turn favoring the expansion of facultative anaerobic clades and hindering enterocyte proliferation [100, 101]. The resulting inflamed intestine perpetuates the production of pro-inflammatory cytokines and inflammogenic microbial metabolites, which affect brain processes and precipitate MDD onset while increasing the likelihood of comorbid conditions [99, 102]. Lastly, cytokines are produced de novo in the brain in response to stress [103–105].

## Psychoneuroimmune Interactions and the Cytokine Hypothesis of Depression

Psychoneuroimmunology studies the reciprocal interactions between behavioral traits and the immune system, mediated by the nervous and endocrine systems [106]. In MDD, increasing evidence suggests that the communication networks existing between the microbiota and the nervous, immune, and endocrine systems lie at the crossroads of psychosocial stress, onset of depressive symptomatology and antidepressant response [107]. Studies suggest anti-inflammatory, endocrine,- and entero-regulatory effects of antidepressants, antidepressant effects of anti-inflammatory medications, and differential responses to antidepressants driven by polymorphisms in inflammation-related genes [108–112]. With regard to the immune players of such communication, cytokines have gained increasing attention over the past 20 years. Cytokines are pleiotropic signaling molecules with immunomodulatory function expressed constitutively and on-demand in the periphery and in the CNS and have been associated in at least a subset of patients with onset, course, and severity of neuropsychiatric disorders, as well as with the response to therapeutic drugs [113–122].

Exposure to psychological stressors primes the immune system towards the creation of a pro-inflammatory environment in the brain, a phenomena called *sterile inflammation*, which prepares the CNS and the body to trigger a potential full-blown immune response [123, 124]. While this program is essential for coping with the stressor and restoring homeostasis, it requires high amounts of energy and has collateral damage potential. In fact, repeated or chronic stress exposure results in a sustained inflammatory milieu in the brain which can lead to the development of MDD and comorbid illnesses [23, 125].

These lines of evidence led to the “cytokine hypothesis” (or “macrophage hypothesis”) of depression, which proposes that cytokines and an out-of-balance brain-immune communication are key MDD milestones [126–130]. This hypothesis is supported by mounting evidence: (a) illnesses characterized by chronic inflammatory responses (e.g., type-1 diabetes and systemic lupus erythematous) are associated with increased depression rates [4, 6], (b) administration of pro-inflammatory cytokines as a therapeutic strategy (e.g., IFNA administration in cancer and hepatitis-C) induces a dose-response depressive symptomatology and molecular features of MDD [131–135], and (c) pro-inflammatory cytokines administration in vivo induces sickness or depressive-like behavior [22, 136]. Lastly, polymorphisms in inflammation-related genes associate with increased MDD susceptibility and differential antidepressant response [25]. These layers of evidence suggest that neuroinflammation is involved in MDD, providing fertile ground to investigate diagnostic and therapeutic opportunities in neuro-immuno-psychiatry.

## Major Depression and Dysregulated Inflammatory Pathways

**Fig. 1:**
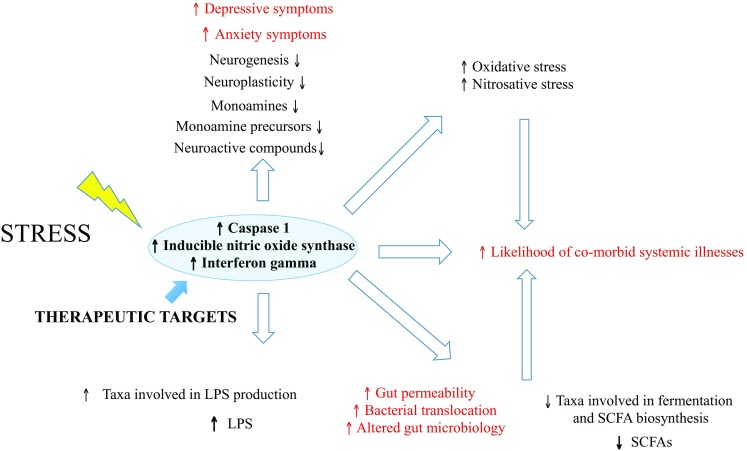
Major depression and dysregulated inflammatory pathways

Psychoneuroimmunology research has highlighted that at least a subgroup of MDD patients present with a systemic low-grade chronic inflammatory profile underlined by increased T cell, monocytic, microglial, and astrocytic activation [23, 24, 137, 138]. This is characterized by increased Th1 cytokines such as IL1, IL2, IL6, TNF, and IFNG, decreased Th2 cytokines such as IL4 and IL10, and decreased regulatory T cells [128, 139–144]. The resulting skewed inflammatory balance triggers multi-level dysfunctions, such as metabolism, neurotransmission, gut microbiome, and neurogenesis alterations [137, 145, 146]. Accordingly, the neurotrophic hypothesis of depression suggests that MDD patients have inflammation-driven decreased neurogenesis, which leads to atrophy of brain areas such as the hippocampus and the prefrontal cortex [147–150]. Not surprisingly, pro-inflammatory cytokines and increased glucocorticoids production downregulate neurotrophins (such as brain derived- and nerve-growth factor) and neurogenesis during and following stress, while antidepressants reverse such decreases [151, 152]. The gut microbiome is also involved in regulating neuroplasticity and neurogenesis; germ-free mice display altered neurogenesis and BDNF expression in the dentate gyrus, while antibiotic treatment impairs neurogenesis [74, 153, 154] (Fig. 1).

## Cytokine Signaling and Nitrosative Stress

Oxidative stress is involved in MDD pathophysiology [155]. Stress exposure leads to ROS upregulation via cytokine-induced NOS2 induction, an event that heightens the overall oxidative stress, activating a feedback loop (co-activation state) that produces more cytokines [138]. Oxidative stress is characterized by the generation of ROS, which contributes to protein and DNA damage, and can result in irreversible brain function changes, leading to neurodegeneration and cognitive impairments [156]. Oxidative processes are gaining attention in psychiatry, since an expanding body of research suggests the involvement of these pathways in MDD [24, 40, 138, 157–159].

The involvement of oxidative and nitrosative stress in MDD is confirmed by the increased oxidative (such as NO, arachidonic acid, malondialdehyde, and 8-hydroxy-2-deoxyguanosine) and nitrosative (such as immunoglobulin (M IgM)- antibodies directed against phosphatidylitol and nitro-bovine serum albumin) stress markers in MDD patients, together with decreased levels of antioxidants (such as vitamins C and E) [160–164]. Interestingly, the concentration of oxidative stress markers correlates with depression severity and chronicity, as well as with antidepressant response [40, 138, 161, 165]. Accordingly, some antioxidant compounds have antidepressant properties, and antidepressants (such as paroxetine) partially reverse oxidative damage by enhancing the protective antioxidant status following stress [158, 166–168].

Of crucial importance for this work, the NO system is being investigated in MDD, because NO levels are increased in MDD and in animal models of stress, while NO inhibition has antidepressant effects (discussed in detail below) [37, 164, 169–171]. Increased levels of oxidative and nitrosative molecules can easily damage neurons, since they are particularly vulnerable to free radicals [172]. Moreover, the brain presents lower concentrations of antioxidants compared to other organs, making it more susceptible to free radicals [160]. Unsurprisingly, some areas (i.e., the subfields *Cornu Ammonis* (CA)1) and CA4) of the hippocampus (a brain region involved in mood regulation and adult neurogenesis) are the most sensitive to oxidative damage [24].

## The Role of Caspase 1 in MDD

As mentioned above, stress triggers “sterile inflammation,” initiated by endogenous danger signal recognition, termed damage-associated molecular patterns (DAMPs), by glial cells, macrophages, and oligodendrocytes [124, 181, 182]. DAMPS are nuclear, cytosolic, mitochondrial, or extracellular molecules normally hidden from the immune system that upon activation are exposed and released in the extracellular space, where they stimulate an immune activation [124, 183]. In line with this understanding, increased levels of DAMPs have been found in rodent blood and hippocampus following stress exposure [103, 184].

Once released in the extracellular space, DAMPs function as alarm signals, alerting immune cells through pattern recognition receptors, to get ready for a potential full-blown immune response [182, 185, 186]. It has been hypothesized that such processes could represent an adaptive characteristic of the acute stress response; for example, if an animal were running away from a predator and were wounded during the chase, it might have better chances of surviving if its immune system were primed and ready to respond [187]. Another theory, one that places this mechanism in a modern context, suggests that such stress responses are activated when an individual is exposed to social evaluation, rejection, isolation, exclusion or conflict, possibly due to the potentially physically harmful significance of such social situations throughout history [188].

Together, DAMPs activation and release induce the transcriptional upregulation of a number of immune genes, such as IL1B, IL6, and TNF. This results in the creation of a pro-inflammatory milieu in the brain and periphery, and in the activation of the afferent nerves, which in turn leads to de novo production of pro-inflammatory cytokines in the brain and culminates with the onset of depressive-like behavior [22, 136, 189].

Further, DAMP activation results in the assembly of inflammasomes [186, 190] A peculiar role is played by the NLRP3 inflammasome, that consists of the NLRP3 protein, the adaptor apoptosis-associated speck-like protein containing a CARD (ASC), and the cysteine-protease CASP1 [47]. Upon inflammasome assembly, the inactive procaspase 1 zymogen is proteolitically cleaved into the enzymatically active heterodimer [191, 192]. In turn, activated CASP1 cleaves pro-IL1B and pro-IL18 into their mature, releasable, bioactive isoforms [47, 193]. Increased circulating levels of IL1B activate the HPA axis, which increases glucocorticoids production. [72]

CASP1 and NLRP3 transcripts and their protein products are increased in peripheral blood mononuclear cells (PBMC) from MDD patients compared to controls, while antidepressants decrease such hyperactivity [61]. Similarly, IL1B and IL18 are increased in MDD, and their levels correlate with the severity of depression [61] (Table 1). Correspondingly, antidepressants decrease IL1B levels [109].

**Table 1:** Clinical evidence of CASP1 involvement in MDD

Clinical evidence	Reference
Increased CASP1 and NLRP3 transcription in PBMC (peripheral blood mononuclear cells) from MDD patients. Increased NLRP3 protein levels in PBMC from MDD patients. Increased IL1B and IL18 in serum from MDD patients which positively correlate with BDI (Beck Depression Inventory) score. Antidepressant treatment decreased NLRP3 and CASP1 transcription in PBMC from MDD patients. Antidepressant treatment decreased IL1B and IL18 in serum from MDD patients.	[61]
IL18 is increased in MDD patients.	[62, 63]
IL18 is increased in patients with panic disorder.	[63]
IL18 promoter variants (rs187238 and rs1946518) associate with higher IL18 transcription and increased susceptibility to MDD in patients exposed to stressful events.	[64]
Polymorphisms in the IL33 gene (rs11792633 and rs7044343) moderate the correlation between history of childhood abuse and recurrent depression in women.	[65]
Patients with recurrent depression have higher peripheral IL33	[65]

*Casp1*^−/−^ mice display decreased depressive- and anxiety-like behaviors, while being protected by the exacerbation of depressive-like behavior following chronic stress [19, 173]. Similarly, minocycline-treated mice display resilience in developing depressive-like behavior following stress, and this effect is accompanied by the expansion of bacterial clades with anti-inflammatory properties, which could help explain minocycline’s antidepressant effects [19] (Table 2).

**Table 2:** Pre-clinical evidence of CASP1 involvement in animal models of MDD

Pre-clinical evidence	Reference
Chronic unpredictable mild stress (CUMS) increases PFC (prefrontal cortex) CASP1 activation and NLRP3 and IL1B transcription and protein level. Antidepressant treatment decreases PFC NLRP3 protein level and IL1B transcription and protein level.	[173]
LPS-induced depressive-like behavior increases brain CASP1, NLRP3, and ASC transcription, and IL1B transcription and protein level. Pre-treatment with an NLRP3 inhibitor (Ac -YVAD-CMK) ameliorates depressive-like behavior.	[174]
CUMS increases hippocampal and serum Il1b and increases hippocampal CASP1 activity and NLRP3 and ASC protein levels. Pretreatment with the NLRP3 inflammasome inhibitor VX-765 decreases serum and hippocampal IL1B protein levels and decreases depressive-like behavior.	[175]
*CASP1*^−/−^ mice display decreased depressive- and anxiety-like behaviors, while being protected by the exacerbation of depressive-like behavior following chronic stress. The CASP1 inhibitor minocycline prevents the exacerbation of depressive-like behavior following stress. Minocycline triggers the expansion of bacterial populations with anti-inflammatory effects.	[19]
CUMS increase hippocampal IL1B. IL1R^−/−^ mice do not display CUMS-induced behavioral or neuroendocrine changes. IL1R^−/−^ mice do not display CUMS-induced decreases in neurogenesis. IL1B exogenous administration mimics CUMS-induced depressive-like symptoms.	[176]
Stress and Il1b administration suppress hippocampal cell proliferation. IL1R1 blockade blocks the antineurogenic effects of stress.	[177]
*IL18*^−/−^ mice display decreased depressive- and anxiety-like behaviors.	[178]
IL18 is involved in stress-induced microglial activation while contributing to dopaminergic degeneration.	[179, 180]
Acute stress increases IL33 expression in the paraventricular nucleus of the hypothalamus and in the prefrontal cortex.	[65]

*CASP1*^−/−^ mice have the same behavioral and inflammatory responses to systemic lipopolysaccharide (LPS) administration as wild-type (wt) mice, but are resistant to the development of depressive-like behavior and to pro-inflammatory cytokines increase following intracerebroventricular LPS administration [194]. Moreover, *CASP1*^−/−^ mice are resistant to lethal LPS doses and have decreased levels of inflammation-induced brain and systemic transcription [195–197]. Significantly for this review, CASP1 and the NLRP3 inflammasome are involved in the development of depressive-like behavior in stress models and are increased in MDD [61, 173]. At the same time, pathological shifts in gut microbiota composition and leaky gut trigger an increase in pro-inflammatory signaling, which increases the risk of developing depressive symptomatology and comorbid illnesses [198]. Such evidence has led to the formulation of the microbiota-inflammasome hypothesis of major depression and comorbid systemic illnesses [58]. This hypothesis suggests that pathological gut microbiome shifts upregulate pro-inflammatory pathways exacerbating depressive symptomatology and increasing the likelihood of developing comorbid conditions [58].

### Interleukin-1B (IL1B)

IL1B binds to the interleukin-1 receptor (IL1R1), which results in the activation of many acute-phase inflammation genes, such as NOS2, IL6, and cyclooxygenase type 2 [192, 199]. Recently, it was suggested that NLRP3 inflammasome activation mediates IL1B orchestrated inflammation (that results in depressive-like behavior) in the prefrontal cortex following stress, and that fluoxetine reverses such changes [173, 175]. Accordingly, mice lacking the IL1 receptor are resistant to developing depressive-like behavior following chronic stress while being protected against the decrease in neurogenesis observed in wt mice following stress [176, 177].

### Interleukin-1A (IL1A)

IL1A shares features with IL1B and is an equally potent pro-inflammatory cytokine [207]. However, IL1A also presents differences to IL1B. For example, unlike the IL1B precursor which is not active, both the pro-IL1A and the cleaved IL1A are active ligands of the IL1R1 [208]. Moreover, while IL1B is released, IL1A can be secreted or membrane-bound, although the factors that control such translocation have not been fully elucidated yet [207, 209]. Finally, while IL1B is produced on-demand in immune cells, IL1A is constitutively expressed in a variety of cell types but can be produced by immune cells in response to insults [210]. Interestingly, IL1A-mediated activation of p38-MAPK inhibits NR3C1 function, suggesting that the mechanism conferring glucocorticoid resistance in MDD could be associated with IL1A [211]. To the best of our knowledge, no studies have investigated anxiety- and depressive-like phenotypes in *IL1A*^−/−^ mice.

### Interleukin-18 (IL18)

IL18 is a prototypical Th1 cytokine for its ability to stimulate IFNG activity, and it is expressed in macrophages and dendritic cells [212]. Circulating IL18 increases during stress and in response to HPA axis activation [213]. IL18 binds to the IL18 receptor (IL18R) activating p38-MAPK, c-Jun N-terminal kinase, and NFKB1 cascades, which potentiate antimicrobial and antiviral immunity [214, 215]. Although IL18 is known for its ability to promote both Th1- and Th2-related inflammatory responses, its predominant role in enhancing Th1 activity makes this cytokine a candidate therapeutic target in Th1-related inflammatory and autoimmune diseases, including MDD [212].

IL18 is increased in MDD and in panic disorder [62, 63]. IL18 gene promoter variants (rs187238 and rs1946518) associate with higher IL18 transcription and increased MDD susceptibility in patients exposed to stressful events. *IL18*^−/−^ mice have decreased IFNG production and impaired natural killer cell activity and abnormal Th1 responses [216]. Moreover, *IL18*^−/−^ mice display decreased depressive- and anxiety-like behavior, as well as gene expression changes across various brain regions [178, 217]. In addition, immobilization stress in mice induces pro-IL18 via ACTH and a superoxide-activated CASP1 pathway [218]. Given that IL6 is not induced in response to stress in *IL18*^−/−^ mice, it seems that IL18 mediates stress-induced IL6 upregulation [218]. Lastly, IL18 is involved in stress-induced microglial activation in rodents while contributing to dopaminergic degeneration [179, 180].

### Interleukin-33 (IL33)

IL33 has alarmin and transcription factor roles and triggers predominantly Th2 responses (such as the induction of IL4, IL5, IL13, and anti-inflammatory gene expression) [221]. Like other members of the IL1 family, IL33 can be beneficial or detrimental, depending on its spatio-temporal expression. IL33 is constitutively expressed and localized in the cytoplasm. However, if a barrier is breached and IL33 is released from destroyed cells, it acts as an alarmin upon binding the IL33 receptor (ST2) [222]. The signaling cascade in response to ST2 activation modulates hundreds of genes with a pattern that resembles that of IL1R1 activation [223].

Two single nucleotide polymorphisms in the *IL33* gene (rs11792633 and rs7044343) moderate the correlation between history of childhood abuse and recurrent depression in women [65]. Moreover, patients with a history of recurrent depression have greater peripheral levels of IL33 and IL1B [65]. Finally, IL33 is expressed in the paraventricular nucleus of the hypothalamus and in the prefrontal cortex of rats exposed to acute stress, suggesting that stress induces IL33 expression in those brain regions [65].

## The Role of Inducible Nitric Oxide Synthase in MDD

NO is a small intercellular and intracellular signaling molecule with a very short half-life (3–6 s) that freely diffuses across cell membranes. NO plays important roles in the brain modulating pathways such as neurogenesis, neurotransmission, synaptic plasticity, learning, and pain [224]. NO also regulates emotional and cognitive processes, suggesting that it could be involved in the etiology of MDD and anxiety disorders [225]. Three isoforms of the NOS enzyme produce NO: NOS2*,* neuronal (NOS1), and endothelial (NOS3), all of which have specific spatio-temporal patterns of regulation. In this review, we will focus on the inducible isoform since it is considered the most relevant to MDD.

Over the past two decades, several lines of evidence have brought NO and specifically the NOS2 isoform to the forefront in psychiatry: (a) the levels of NO and its metabolites are increased in MDD patients and suicide attempters compared to controls [171, 200, 201], (b) *NOS2* transcription is increased in the peripheral blood of patients with recurrent depressive disorder [202], (c) a polymorphism (-1026C/A) in the *NOS2* promoter associates with recurrent depressive disorder risk [203], (d) IgM against NO adducts are elevated in MDD patients, suggesting that the protein damage created by NO results in the formation of immunogenic peptides, that in turn activate an autoimmune-like response [204, 205], (e) the selective serotonin reuptake inhibitor paroxetine is a NOS2 inhibitor [206, 226], (f) adjuvant NOS2 inhibition enhances the efficacy of serotonergic antidepressants [169], and (g) NOS2 is increased in the hippocampus and cerebral cortex in mice following stress, and NOS2 inhibition results in antidepressant-like effects in rodents [38, 219, 220] (Tables 3-4).

**Table 3:** Clinical evidence of NOS2 involvement in MDD

Clinical evidence	Reference
Increased plasma nitric oxide (NO) metabolites in suicide attempters. Increased plasma NO metabolites in depressed suicide attempters.	[171]
Increased plasma NO metabolites in suicide attempters. Higher plasma NO levels were related to lower suicide lethality and lower depression severity.	[200]
Increased plasma nitrate concentration in MDD patients.	[201]
Increased *NOS2* transcription in peripheral blood of MDD patients.	[202]
The polymorphism (-1026C/A) in the *NOS2* promoter is associated with the risk of recurrent depressive disorder.	[203]
IgM levels against NO adducts are elevated in MDD patients, suggesting an autoimmune-like response.	[204, 205]
The antidepressant paroxetine is a NOS2 inhibitor.	[206]

**Table 4:** Pre-clinical evidence of NOS2 involvement in animal models of MDD

Pre-clinical evidence	Reference
NOS2 inhibitors augment the efficacy of serotonin reuptake inhibitors in the forced swim test.	[169]
NOS2 is increased in the hippocampus and cerebral cortex following stress.	[38]
NOS2 inhibition results in antidepressant-like effects in rodents.	[219]
The dopamine reuptake inhibitor bupropion modulates the NO system.	[220]

The architecture of the *NOS2* promoter region suggests that this gene has a tight and complex pattern of transcriptional control since it is rich in positive and negative regulatory regions, and it is responsive to many transcription factors, cytokines, and bacterial by-products [29]. NOS2 is synthesized on-demand in macrophages and microglia [227]. In fact, whereas there is no detectable physiological NOS2 expression in the brain, a profound transcriptional upregulation of the *NOS2* gene can be observed in response to traumatic events such as ischemia and systemic inflammation, most likely through activation of the *NOS2* promoter by inflammation-related molecules [29, 39, 196, 228, 229]. Following induction, NOS2 produces NO continuously until the proteasome degradation pathway inactivates the enzyme [230].

Several studies have targeted the NO system in pre-clinical MDD research, yielding promising results. For example, NO decreases norepinephrine production, decreases nitrate and nitrite levels in the hippocampus and cerebral cortex, and decreases serotonin turnover in the frontal cortex [231–233]. Moreover, NO inhibits the dopamine transporter, indirectly increasing the availability of inter-synaptic dopamine [234]. Finally, several molecules such as bupropion (a norepinephrine-dopamine reuptake inhibitor), venlafaxine (a serotonin-norepinephrine reuptake inhibitor), mementine (an NMDA receptor antagonist), and berberine (a plant alkaloid), all of which produce antidepressant-like effects, modulate this signaling pathway [235].

It is accepted that anaerobic bacteria in the gut prevent the expansion of facultative anaerobic bacteria, at least partially by limiting the host-mediated production of oxygen and nitrate [236]. Antibiotic-mediated disruption of the gut microbiota increases the production of host nitrate in the gut [237]. This allows an expansion of the facultative anaerobic Enterobacteriaceae, which includes potentially pathogenic gram-negative bacteria, such as *Escherichia coli* (this effect is likely not to be limited to *E. coli*, although the latter has been the focus of investigation to date). These bacteria produce the virulence molecule LPS, which triggers depressive-like behavior and increases serotonin degradation in the brain [237, 238]. This alteration is mediated by NOS2; therefore, its inhibition prevents *E. coli* overgrowth [237]. Therefore, rectifying aberrant NO signaling could have a therapeutic role in altered gut microbiology-induced depressive symptoms [239]. Accordingly, stimulation of colonic epithelial cancer cells by IFNG induces NOS2-mediated NO production, while butyrate (one of the main anti-inflammatory short chain fatty acids (SCFAs)) blunts NO production [237]. This result suggests that a diet rich in substrates for SCFAs production could have antidepressant-like effects via its repercussions on gut microbiome composition and inflammatory processes. Together, these findings suggest that modulation of the NO system could represent a useful approach in treating MDD and in keeping of a healthy gut microbiome.

## The Role of Interferon-Gamma in MDD

IFNG is a pleiotropic soluble cytokine which orchestrates cellular programs via transcriptional and translational gene control. IFNG is produced by immune cells such as lymphocytes, cytotoxic lymphocytes, B cells, and antigen-presenting cells [240, 241]. The IFNG receptor (IFNGR) is expressed on almost all cell types, and its activation triggers the janus kinase 1 and 2 (JAK1/2) signal transducer and activator of transcription 1 (STAT1) pathway, as well as additional pathways, such as the extracellular-signal-regulated-kinase 1/2 (ERK1/2) [242, 243]. Activation of the IFNGR results in the transcription of genes with IFNG-stimulated response elements (ISREs) within their promoter region until STAT1 dissociates following complete dephosphorylation within 1–2 h [244, 245]. The genes transcribed in response to IFNGR activation are at least 200, together with many micro RNAs and long non-coding RNAs [246] (for a database see [247]). At the same time, after IFNGR stimulation, the secondary transcription factors IRF1, IRF2, and interferon consensus sequence binding protein are upregulated. This in turn results in the transcriptional induction of a subset of inflammatory-related genes such as *NOS2* (stimulated by IRF1) and guanylate-binding protein. Finally, IFNG can activate and be activated by CASP [248–251].

Ex vivo PBMC from MDD patients display increased IFNG and neopterin production upon stimulation, as well as decreased tryptophan bioavailability [252]. Nevertheless, IFNG transcriptional levels (together with those of TNF) in patients with multiple sclerosis correlate with the severity of the depressive symptomatology during flare-ups [253]. At the same time, most categories of antidepressants suppress the IFNG/IL10 ratio through suppressing IFNG and stimulating IL10 [254, 255]. These findings (Table 5) suggest that MDD patients have increased systemic IFNG and neopterin production by activated T cells and macrophages. This could be responsible for an upregulation of the enzyme IDO1 (since the latter presents 2 ISREs at the promoter region that lead to maximum promoter activity) and consequent tryptophan depletion through upregulation of the kyneurine/tryptophan pathway, events that decrease serotonin availability and increase the toxic metabolite kyneurine [252, 258–260]. Accordingly, a polymorphism (CA repeat, rs3138557) in the *IFNG* gene correlates with lower serum tryptophan and 5-hydroxindolacetic acid (the main metabolite of serotonin) and higher levels of kyneurine, suggesting that carriers of the CA allele might be more susceptible to developing MDD [256]. Similarly, the presence of the high producer T allele +874(T/A) polymorphism (rs2430561) associates with increased IDO1 activity [257]. Interestingly, IFNG signaling drives Th1 development [261, 262]; therefore, early increased signaling of IFNG by traumatic events could be involved in the Th1/Th2 shift towards Th1 in MDD [141].

**Table 5:** Clinical evidence of IFNG involvement in MDD

Clinical evidence	Reference
Ex vivo PBMC from MDD patients display increased IFNG production upon stimulation.	[252]
Transcriptional levels of IFNG correlate with depressive symptomatology in multiple sclerosis patients.	[253]
The antidepressants clomipramine, sertraline, and trazodone suppress IFNG production.	[254, 255]
A polymorphism in the *IFNG* gene (CA repeat, rs3138557) correlates with lower serum tryptophan and higher kyneurine increasing MDD likelihood.	[256]
The high producer T allele + 874(T/A) polymorphism (rs2430561) in the *IFNG* gene has been associated with increased IDO1 activity and increased MDD likelihood.	[257]

*IFNG*^−/−^ mice do not show developmental defects but present compromised immune responses and increased susceptibility to infections [263]. With regard to their behavior, *IFNG*^*−*/−^ mice display decreased anxiety- and depressive-like behaviors as well as heightened emotionality in several paradigms [264–266]. These behaviors are underlined by (a) increased serotonergic and noradrenergic activity (i.e., greater metabolite accumulation) in the central amygdaloid nucleus, together with (b) increased baseline plasma corticosterone, (c) decreased neurogenesis in the hippocampus, and (d) decreased levels of nerve-growth factor in the prefrontal cortex, suggesting that IFNG modulates anxiety and depressive states and is involved in CNS plasticity [264, 265]. On the other hand, while IFNG deficiency does not confer resistance to a chronic stress regimen in mice, it attenuates monoamine, corticoid, and cytokine alterations in response to stressors [264] (Table 6).

**Table 6:** Pre-clinical evidence of IFNG involvement in animal models of MDD

Pre-clinical evidence	Reference
*IFNG*^*−*/−^ mice display decreased anxiety- and depressive-like behaviors as well as heightened emotionality.	[264–266]
*IFNG*^*−*/−^ mice display increased serotonergic and noradrenergic metabolite accumulation.	[264, 265]
*IFNG*^*−*/−^ mice display increased plasma corticosterone levels.	[264, 265]
*IFNG*^*−*/−^ mice display decreased hippocampal neurogenesis.	[264, 265]
*IFNG*^*−*/−^ mice display decreased levels of nerve growth factor in the prefrontal cortex.	[264, 265]
*IFNG*^*−*/−^ mice have attenuated monoamine, corticoid, and cytokine alterations in response to stressors.	[264]

IFNG signaling promotes leaky gut and bacterial translocation. In fact, in vitro experiments have highlighted that low-dose IFNG dramatically increases the translocation of opportunistic pathogens, and high-doses disrupt tight junctions [267]. Lastly, IFNG levels affect the representation of specific bacterial species while being up- or downregulated by specific commensals [97]. For example, the degradation of tryptophan to the metabolite tryptophol inhibits IFNG production, while IFNG levels dictate the presence and expansion of specific bacterial taxa [97]. Given this evidence for an involvement of IFNG in pathways relevant to depressive symptoms and gut dysbiosis, targeting IFNG and/or its receptor could hold potential in the quest for novel MDD therapies.

## Conclusions and Future Directions

Convergent pre-clinical and clinical evidence points towards an involvement of central and peripheral inflammatory pathways and the gut microbiome in the response to psychological stressors and in the onset, treatment, and remission of MDD. Future randomized controlled trials should investigate the safety and efficacy of decreasing CASP1-, NOS2,- and IFNG-mediated pathways in MDD patients. Reduced activity of those pro-inflammatory mediators could be achieved via pharmacological inhibition or gut microbiome manipulation. The latter approach can involve diet, probiotics supplementation, and fecal microbiota transplantation. This could lead to the development of novel antidepressant strategies acting upon the dysregulated inflammatory milieu observed in MDD. Because inhibiting such pathways might hinder physiological immune processes, particular care should be taken when developing immunomodulatory and gut microbiota-directed therapies.
